# Speed impairs attending on the left: comparing attentional asymmetries for neglect patients in speeded and unspeeded cueing tasks

**DOI:** 10.3389/fnhum.2012.00232

**Published:** 2012-08-21

**Authors:** Kristie R. Dukewich, Gail A. Eskes, Michael A. Lawrence, Mary-Beth MacIsaac, Stephen J. Phillips, Raymond M. Klein

**Affiliations:** ^1^Department of Psychiatry, Dalhousie UniversityHalifax, NS, Canada; ^2^Department of Psychology, Dalhousie UniversityHalifax, NS, Canada; ^3^Department of Medicine, Neurology, Dalhousie UniversityHalifax, NS, Canada

**Keywords:** attention, spatial cueing, temporal order judgments, unilateral neglect

## Abstract

Visuospatial neglect after stroke is often characterized by a disengage deficit on a cued orienting task, in which individuals are disproportionately slower to respond to targets presented on the contralesional side of space following an ispilesional cue as compared to the reverse. The purpose of this study was to investigate the generality of the finding of a disengage deficit on another measure of cued attention, the temporal order judgment (TOJ) task, that does not depend upon speeded manual responses. Individuals with right hemisphere stroke with and without spatial neglect and older healthy controls (OHC) were tested with both a speeded RT cueing task and an unspeeded TOJ-with-cuing task. All stroke patients evidenced a disengage deficit on the speeded RT cueing task, although the size and direction of the bias was not associated with the severity of neglect. In contrast, few neglect patients showed a disengage deficit on the TOJ task. This discrepancy suggests that the disengage deficit may be related to task demands, rather than solely due to impaired attentional mechanisms *per se*. Further, the results of our study show that the disengage deficit is neither necessary nor sufficient for neglect to manifest.

## Introduction

Visuospatial neglect is a condition whereby people are deficient at attending to or noticing the contralesional side of space when the deficiency cannot be explained by primary sensory or motor deficits (Heilman and Valenstein, 1979). As such, neglect is often described as a cognitive disorder of attention (Bisiach et al., 1979; Posner and Petersen, 1990; Karnath et al., 2002b). The condition is more likely to manifest in people with right rather than left hemisphere lesions, and has been associated with lesions in a variety of brain regions and white matter networks, including anterior frontal lobe, the parietal lobe and tempo-parietal junction, superior temporal gyrus as well as subcortical areas (Mesulam, 1981; Vallar and Perani, 1986; Leibovitch et al., 1998; Vallar, 2001; Karnath et al., 2002a,b, 2004; Mort et al., 2003; Bartolomeo et al., 2007).

One of the most popular experimental paradigms for studying attention and neglect is the exogenous spatial cueing task developed by Posner and colleagues in the late 1970s (Posner, 1978, 1980; Posner et al., 1980). The task involves presenting participants with a central fixation flanked by two peripheral boxes. A cue is presented in one of the two peripheral locations, followed by a target presented in one of those locations; the cue may or may not predict the location of the target. When the time interval between the cue and the target is short (approximately 250 ms), participants are typically faster to respond to the target when it appears at the same location as the cue (a “valid” or “cued” trial) compared to when it appears at the opposite location (an “invalid” or “uncued” trial)—an effect referred to as facilitation (Posner and Cohen, 1984). The spatial cueing task has been exploited to examine various aspects of spatial attention and has allowed the description of several general attention-related effects (cf. Posner et al., 1985), as well as specific effects related to various neuropsychological and psychiatric disorders (cf. Maruff et al., 1995; Townsend et al., 1996).

Posner used the spatial cueing paradigm to help develop a model of orienting involving three distinct operations: attention first disengages from its current focus, it then shifts toward the new target location, and finally attention engages the new target (Posner et al., 1982, 1984). Posner and colleagues were the first to use the spatial cueing paradigm to investigate the effects of parietal lobe lesions and reported that after left or right parietal lobe damage, while individuals were able to benefit from cues provided on the same side as the target, they were disproportionately slower to respond to targets presented on the contralesional side of space following an ispilesional cue as compared to the reverse (Posner et al., 1982). Posner and others have characterized this pattern (increased cost for contralesional targets following ipsilesional cues) as a difficulty disengaging attention from the patient's “good” field in order to deal with a target presented to the “poor” field, and have thus christened the effect a *disengage deficit* (Posner et al., 1982; Rastelli et al., 2008).

Losier and Klein (2001)^1^ conducted a meta-analysis of the literature dealing with the disengage deficit to reveal several important characteristics of the effect. As with neglect itself, the disengage deficit is greater in patients with right compared to left hemisphere damage. In patients with right hemisphere damage, the disengage deficit is greater using shorter cue-target onset asynchronies (CTOAs; i.e., less than 550 ms) compared to longer CTOAs (Losier and Klein, 2001). However, patients with left hemisphere damage tend to have a relatively stable disengage deficit across cue-target intervals. There is some evidence that patients with damage to one hemisphere who fail to show clinical signs of neglect can exhibit a disengage deficit; however, the disengage deficit is significantly larger in patients *with* neglect (cf. Posner et al., 1984). Additionally, the size of the disengage deficit is related to neglect severity, such that patients with more severe neglect tend to have larger disengage deficits (e.g., Baynes et al., 1986; Morrow and Ratcliff, 1988; Farah et al., 1989; D'Erme et al., 1994; Egly et al., 1994; Losier and Klein, 2001; Snyder and Chatterjee, 2006; Bonato et al., 2009; Schindler et al., 2009; Olk et al., 2010), although this relationship has not always been found (Posner et al., 1984; Sacher et al., 2004; Sieroff et al., 2007). This effect is amplified in right-hemisphere neglect patients compared to left-hemisphere neglect patients. Recently, Rastelli et al. (2008) have shown that the disengage deficit is greater when the cue remains on screen for the entire trial compared to when the cue is removed before the target appears, suggesting the disengage deficit is stronger for objects than it is for locations.

The current interpretation of the disengage deficit implies something general about the way that neglect manifests; once attention is captured by a cue in the good field, targets presented to the poor field have particular difficulty generating disengagement from this cue. However, to our knowledge, the effect has only been studied using variants of Posner's spatial cueing paradigm. If the disengage deficit is actually about attention, then it should generalize to other paradigms sensitive to attentional cueing. One candidate task for testing this hypothesis is the temporal order judgment (TOJ) paradigm. In a conventional TOJ task two stimuli are presented in rapid succession, e.g., one on the left and one on the right side. The order of side of first presentation, left or right, varies across trials, along with the interval, or stimulus onset asynchrony (SOA), between the stimulus onsets. To avoid response biases, participants can be asked to report which item was presented first using a stimulus characteristic (e.g., color or orientation) rather than stimulus location (Spence et al., 2001). Typically, left-first trials are coded with negative SOAs while right-first trials are coded with positive SOAs; the likelihood of reporting the item on the right as appearing first is calculated and plotted per SOA. The SOA at which the likelihood for reporting right-first is 50% is considered the point at which participants subjectively experience the two events as occurring at the same time. This SOA is referred to as the point of subjective simultaneity (PSS). Normal observers are usually very accurate, and under neutral conditions without cueing the PSS averages around 0, indicating no spatial asymmetry. Judgments in this task are also influenced by spatial cueing (Stelmach and Herdman, 1991; Shore et al., 2001), such that presenting a cue on either the left or right side prior to presenting the test stimuli can impact which item participants perceive as occurring first. This shift in the PSS is presumed to reflect a perceptual change due to the drawing of attention to the cued side, with corresponding earlier arrival at a temporal comparison stage (Stelmach and Herdman, 1991; Shore et al., 2001). The TOJ task has been used to reveal visual spatial attention asymmetries in individuals with extinction and/or spatial neglect, and a number of investigators have reported a shift in the PSS under neutral conditions without cueing such that the stimulus on the contralesional side must temporally lead the stimulus on the ipsilesional side in order for the two to be perceived as simultaneous (Rorden et al., 1997; Robertson et al., 1998; Baylis et al., 2002; Berberovic et al., 2004; Sinnett et al., 2007; but see Dove et al., 2007).

While Posner's speeded RT cueing task and the unspeeded TOJ-with-cueing task share similar characteristics in spatial cuing (e.g., Eskes et al., 2007) that suggest neglect patients will show a disengage deficit in both tasks, there are several differences and reported dissociations between the tasks which may impact the results (e.g., Neumann et al., 1993; Miller and Schwarz, 2006). TOJ tasks *can* require participants to make a speeded response (cf. Heath, 1984; Shore et al., 2001), but because the primary measure is related to accuracy and SOA rather than speed, this requirement is unnecessary and the authors know of no published reports in patients using a speeded TOJ task. In the speeded RT cueing task, the actions of attention are inferred by RTs, and so speeded responding cannot be avoided. Table 1 summarizes the task characteristics for the TOJ and RT tasks.

**Table 1:** **Summary comparison of the characteristics of the two experimental tasks**.

**Characteristic**	**RT task**	**TOJ task**
Sensitive to spatial cueing	Yes	Yes
Speeded response required	Yes	No
Disengage deficit	Yes	?

Some evidence that the disengage deficit may not transfer to a TOJ task was hinted at in Di Pellegrino et al. (1997). Di Pellegrino et al. described a case study of a 65-year-old patient with neglect and extinction following a right-hemisphere stroke. The patient was asked to report the identity of two target letters presented asynchronously, one on either side of a central fixation. If the patient's neglect and extinction were due to a disengage deficit, one would expect that contralesional targets would be less likely to be identified correctly when an ipsilesional target was presented first compared to when the ipsilesional target was presented second in the pair. However, the researchers found that the patient was significantly worse at reporting a contralesional target if it was presented within 600 ms of the letter presented on the ipsilesional side of space, and that the deficit was similar in duration and magnitude irrespective of whether the contralesional target was present first or second in the pair. Di Pellegrino et al. explain these results in terms of a competitive model of selective attention, rather than a model that assumes a difficulty in disengaging from ipsilesional objects. In the competitive model, ipsilesional targets are assumed to have a higher weight in terms of capturing selective attention, such that even if they arrive 300–400 ms after a contralesional target they still manage to capture attentional resources and interrupt contralesional processing before contralesional target identity is determined.

The current paper explores whether the disengage deficit is observable in a TOJ-with-cueing task as well as in a speeded RT cueing task that are matched for stimuli and task decision. The goal is to determine whether, when the stimuli (cues and targets) and decision demands of the two task are relatively similar, the disengage deficit is a general phenomenon of neglect, or whether it is specific to the speeded RT cueing task. The TOJ task in the current experiment was conducted using an *unspeeded* response, while the RT task was conducted using standard *speeded* responses. If both tasks produce a disengage deficit, this similarity would provide converging evidence that neglect is a general problem of attentional orienting. However, if the TOJ task fails to produce a disengage deficit, then this difference would suggest that the effect has more to do with speeded responding than attention.

While most cases of neglect involve changes in processing of the contralesional side of space associated with an ipsilesional disengage deficit, there have been some reports of ipsilesional neglect (Kim et al., 1999). Patients with ipsilesional neglect might be expected to show a contralesional disengage deficit. To differentiate these two patterns, we will refer to the ipsilesional disengage deficit predicted by Posner et al. (1984) model of attention as a standard disengage deficit, and a contralesional disengage deficit as a paradoxical disengage deficit. In the current study, only patients with right hemisphere damage were tested and so their attentional deficits, if any, should appear on the left side of space. Therefore a standard (ipsilesional) disengage deficit would represent a rightward bias in attention, while a paradoxical (contralateral) disengage deficit would represent a leftward bias in attention.

It should be noted that both tasks in this study required participants to make a similarly demanding 2-choice, non-spatial discrimination. In the RT tasks participants decided as quickly as possible whether the target was red or blue while in the TOJ task participants decided, with no speed pressure, whether the first of two successively presented targets was red or blue. While the disengage deficit is typically studied using simple detection in the RT cueing task, cueing effects are also obtained in non-spatial discrimination (e.g., color discrimination) tasks in studies of visual orienting with control populations, beginning with Jonides and Irwin (1981). The target discrimination task has been suggested by several investigators for use in the TOJ task, specifically (Spence and Driver, 1994; Spence et al., 2001) to be necessary to avoid the possibility that early facilitation is simply the result of a criterion shift for responding to targets at the cued location (i.e., accepting less evidence from that location than the uncued one). False alarms on catch trials (trials without a target) cannot be used in a simple detection task to distinguish speed-accuracy trade-offs because the false alarms cannot be attributed to the cued or uncued location. Therefore, we have two rationales for our decision to use a color choice judgment for the RT task; (1) we wanted to equate stimulus-response demands with the TOJ task, and (2) we wanted the ability to look at errors in order to create an analogous measure for the disengage deficit.

## Materials and methods

### Participants

Individuals with a right hemisphere stroke were recruited for the stroke groups. Inclusion criteria included medically stable and normal or corrected-to-normal visual acuity. Exclusion criteria included other current psychiatric or neurological disorders, severe aphasia or dementia and color blindness. Individuals were assigned to either the neglect group (NEG) or right hemisphere control group (RHC) based on their performance on the Behavioral Inattention Test (BIT). The criterion for neglect was abnormal performance, as based on the standard clinical cut-off, on at least one subtest of the BIT (please see Table A1 for the scores for each of the BIT subtests for each patient). This criterion for neglect was similar to that used by Sieroff et al. (2007) and adopted in order to increase the sensitivity of the BIT to the presence of neglect, as paper and pencil tests are often less sensitive to the presence of neglect in the post-acute or chronic phase (Friedrich and Margolin, 1993; Mattingley et al., 1994; Deouell et al., 2005; List et al., 2008; van Kessel et al., 2010; Bonato et al., 2012; reviewed in Bonato, 2012). Patients' stroke location was determined by clinical CT report. A summary of clinical, demographic and baseline data for participants in the NEG and RHC groups is presented in Table 2. We also included a control group, referred to as older healthy controls (OHC). These participants had no history of stroke, no signs of dementia, and no visual deficits.

**Table 2:** **Demographic and baseline neuropsychological assessment**.

**Subject code**	**Group**	**Stroke location**	**Time post-stroke (mos)**	**Age (years)**	**Gender**	**Dominant hand**	**Education (years)**	**Visual field deficit**	**Visual extinction**	**Judgment of line orientation**	**Elevator counting**	**BIT total**
1047	NEG	P	25	48	M	L	12	No	No	NA	NA	141
1084	NEG	F,T,P,O, cereb	5	71	M	R	12	Yes	N/A	11	7	129
1085	NEG	F, T, Ins	4	75	M	R	11	Yes	Yes	11	3	130
1086	NEG	F, T, BG	3	38	M	R	10	No	No	12	0	135
1090	NEG	F, T, Ins	2	63	M	R	10	Yes	Yes	10	6	119
1157	NEG	P	3	61	M	L	17	No	No	12	4	136
1159	NEG	T, Thal, IC	2	48	M	R	22	No	N/A	12	7	100
1058	RHC	NA	2	79	M	R	12	No	No	NA	NA	141
1081	RHC	F	3	55	M	R	12	No	No	11	1	135
1082	RHC	BG, Ins	1	44	F	R	12	No	No	12	7	144
1087	RHC	T	3	70	M	R	14	No	No	12	7	138
1160	RHC	F, P	2	54	M	R	14	No	No	12	7	144
1162	OHC	–	–	61	F	R	17	No	No	11	7	146
1163	OHC	–	–	51	F	R	11	No	No	12	7	146
1164	OHC	–	–	37	M	R	24	No	No	12	7	146

NA, Not available; NEG, neglect patient; RHC, right hemisphere control patient; OHC, older healthy control participant; F, frontal lobe; T, temporal lobe; P, parietal lobe; O, occipital lobe; cereb, cerebellum; Ins, insular gyrus; Thal, thalamus; IC, internal capsule; BG, basal ganglia.

### Apparatus and stimuli

**Figure 1:**
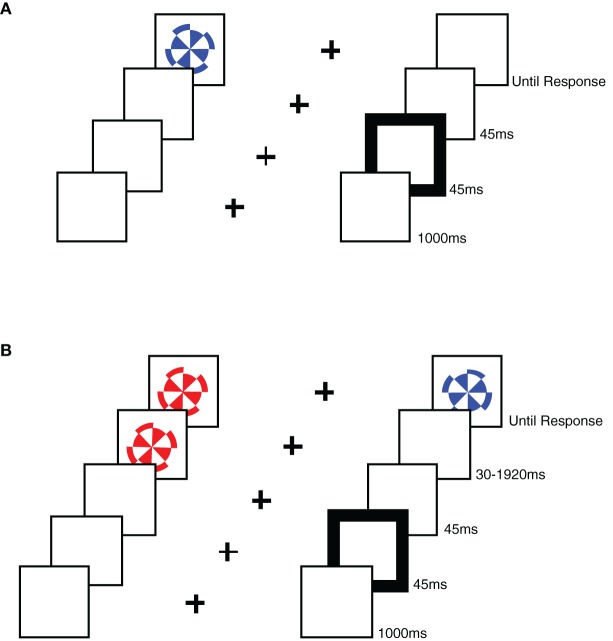
**A schematic representation of the stimuli used in each task. (A)** An example sequence of stimuli in a RT task trial, showing a sequence from the uncued condition. **(B)** An example sequence of stimuli in a TOJ task trial, showing a sequence from the uncued, left-target condition.

Each participant ran in both the RT task as well as the TOJ task. Stimuli were presented on an Apple iMac computer and were programmed using PsyScope Version 1.2.5. We used colored stimuli on a white background. A black fixation cross, measuring 1° × 1° of visual angle (VA), was presented in the center of the screen. Two box outlines in black, each measuring 4° × 4° VA, were positioned to the left and right of fixation offset from center by 4° to each box center. Cues consisted of a 45 ms change in box line-thickness (from one to four points) for one of the two boxes. A stimulus appeared in the center of each box and consisted of a red or blue pinwheel measuring 3° in diameter (see Figure 1). In the RT task, only one pinwheel was presented in one of the two boxes, while in the TOJ task one pinwheel was presented sequentially in each box.

### Procedure

The following tasks were carried out in accordance with the Tri-Council Policy Statement (Canada) and with the approval of the Capital Health Research Ethics Board (formerly the Queen Elizabeth II Health Sciences Center Research Ethics Board). Informed consent was obtained from all the participants.

#### Clinical tests

Standard neuropsychological tests for attention were administered to each participant in addition to the experimental tasks including Judgment of Line Orientation (Benton et al., 1983), the Elevator Counting Task (from the Test of Everyday Attention, Robertson et al., 1996), and the behavioral inattention task (BIT; Stone et al., 1991). The results of these tests are summarized with the patient data in Table 2. Three participants were found to have visual field deficits using the confrontation method; of those three, two showed detection of left-sided items on an in-house computerized perimetry testing task. The third participant with visual field deficits scored normal on the TOJ task. Thus, no individual had a visual field deficit that interfered with testing.

#### RT and TOJ tasks

Participants were seated 57 cm from the computer monitor and instructed to place their first two fingers of their right hand over the “2” and “8” keys of the number pad. The participants were instructed to fixate the central cross, which was on the screen for 1150 ms at the start of each trial. Fixation was visually monitored by the experimenter, and participants were reminded to remain fixated for the duration of the trial whenever they moved their eyes during a trial. A 45 ms cue was presented to one of the boxes (for left or right cue trials) or both boxes (for neutral cue trials) followed by the presentation of the pinwheel(s). Cues appeared at the same location as the target on 50% of trials, and at the opposite location on 50% of the trials.

In the RT task, only one target (a red or blue pinwheel) was presented in one of the peripheral boxes using a CTOA of 90 ms. The target remained visible until a response was made (see Figure 1). Participants ran in 120 trials. Participants were instructed to respond as fast as possible, without losing accuracy, pressing the “2” key if the target was red and the “8” key if the target was blue. Note that these keys were arranged one above the other on the number pad, and were therefore orthogonal to the dimension of spatial cueing (i.e., left vs. right).

In the TOJ task, the time interval between the onset of the cue and the onset of the first target pinwheel (CTOA) was fixed at 90 ms, while SOA between the first pinwheel and the second pinwheel varied, using the following intervals:−1920 ms, −960 ms, −480 ms, −240 ms, −120 ms, −60 ms, −30 ms, 30 ms, 60 ms, 120 ms, 240 ms, 480 ms, 960 ms, and 1920 ms, with negative SOAs indicating left-side first trials. Participants were asked to report the color of the first pinwheel (the target) presented following the cue *without time pressure*, and responses were recorded by pressing the “2” key if the target was red and the “8” key if the target was blue (a manual response was used in the TOJ task in order to better equate it with the RT task). Note that these keys were arranged one above the other on the number pad, and were therefore orthogonal to the dimension of spatial cueing (i.e., left vs. right). Both stimuli remained visible until a response was given. Participants ran in an average of 391 trials, and trials were distributed among the SOAs such that the smallest SOAs were sampled more often than the longest SOAs. The actual percentages used for the selection of SOA on each trial were 13.3% for +/−30 ms, 10% for +/−60 ms, 6.7% for +/−120 ms, +/−240 ms, and +/−480 ms, and 3.3% for +/−960 ms and +/−1920 ms. Although the number of trials per SOA condition varied slightly due to random sampling of SOA condition, on average the proportions achieved were very close to our intentions for both groups. Total trial number varied somewhat due to differences in fatigue level.

### Methods of analysis

In the RT task we used individual mean RTs to calculate two cueing effects (CE = uncued RT minus cued RT) for each participant, one for left-side targets and one for right-side targets. These CEs were then used to create a cueing asymmetry score for each participant. The formula for calculating cueing asymmetry scores is the same as that used to calculate the standard disengage deficit for patients with damage to the right hemisphere: CE_left-side targets_ minus CE_right-side targets_. Positive scores indicate a slower response to targets presented in the left side of space following a right side cue compared to the reverse. This pattern represents a rightward asymmetry, which is the same direction as the standard disengage deficit. Negative scores represent a leftward asymmetry (paradoxical disengage deficit).

We wanted to generate comparable cueing asymmetry scores for both the RT and TOJ tasks. While we have used PSS scores to determine whether there was a cueing effect in the TOJ task (see Eskes et al., 2007), the PSS cannot be coded in terms of the visual field of the target nor in terms of the location of the cue relative to the target, because targets are presented to both fields on every trial in a TOJ study. In order to calculate an analogous cueing asymmetry score for the TOJ task we defined TOJ trials based on “target” side, with a target side referring to the visual field of the item that was presented first. For example, all trials when the left item came up first (i.e., negative SOA trials) were defined as left-target trials. This allowed us to examine error rate as both a function of cueing (uncued vs. cued) and target location, just as in the RT task. We used individual mean error rates to calculate a CE (uncued error rate minus cued error rate) separately for left-side targets and right-side targets. If TOJs are affected in the expected direction by the cues (PSS shift) then errors will necessarily be lower when the cue is presented on the side of the first target (cued) than on the side of the second target (uncued). We then performed the standard cueing asymmetry score subtraction for patients with damage to the right hemisphere (CE_left-side targets_ minus CE_right-side targets_). Positive scores represent a rightward cueing asymmetry, which is in the same direction as the standard disengage deficit, while negative scores represent a leftward cueing asymmetry (paradoxical disengage deficit). For clarity, the meaning ascribed to the direction of these cueing asymmetry scores is congruent with the meaning ascribed to the direction of the cueing asymmetry scores in the RT task.

## Results

### RT task results

We examined error rates and mean RTs for each participant and for the entire set of participants to confirm that each participant was competent at performing the task. One participant in the NEG group had an error rate more than 5 SD from the mean, so he was eliminated from further analysis. To ensure both tasks were easily compared, we also eliminated the same subject from the TOJ analysis described below. All other participants had mean error rates and mean RTs that were within 2 SD of the overall means. The average error rate was 4.2% (SD = 7.1, *n* = 16). A mixed effects ANOVA on errors including cue location and target location as the within-subject variables, and group as the between-subject variable revealed no significant main effects or interactions.

Trials on which participants made erroneous responses (4.2%) and trials on which the participant missed the target (1%) were eliminated from RT analyses. Trials on which RTs were greater than 2 SD above a participant's mean for each condition were considered outliers and were eliminated from subsequent analysis (1.5%). Trials on which RTs were less than 150 ms were also eliminated (0.02%). Mean RTs for the NEG group (*n* = 7), RHC group (*n* = 5), and OHC group (*n* = 3) were 1034 ms (SD = 2224), 1316 ms (SD = 2482), and 494 ms (SD = 194), respectively.

**Figure 2:**
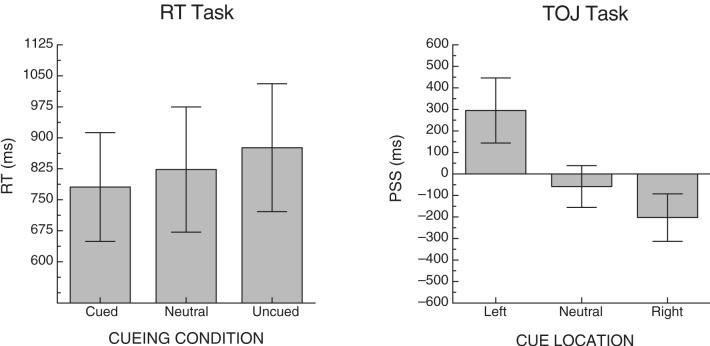
**Mean cueing effects for each of the two tasks across all participants.** Error bars represent +/− the standard error of the mean for each condition.

To ensure that the RT task was effective at producing a cueing effect, mean RTs were calculated by cueing condition collapsed over side. The overall mean for cued, neutral and uncued trials were 781 ms (SD = 510), 823 ms (SD = 587), and 876 ms (SD = 600), respectively (see left graph, Figure 2). A one-way ANOVA revealed a significant effect of cueing condition [*F*(2, 28) = 5.91, *p* < 0.01, MSE = 5753].

**Figure 3:**
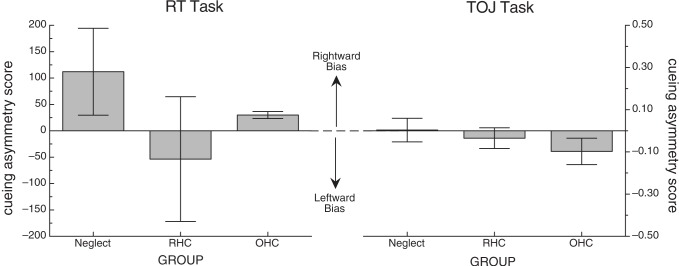
**Mean cueing asymmetry score for each of the two tasks.** Error bars represent +/− the standard error of the mean for each group. In both cases, positive scores represent a rightward bias (the direction of the standard disengage deficit) and negative scores represent a leftward bias.

Individual mean RTs were then used to calculate cueing effects (CEs) and cueing asymmetry scores for left- and right-side targets for each participant (see section “*Methods of Analysis*”, above). From these data, group averages were created. Table 3 includes the descriptive statistics by group for left- and right-side target CEs and cueing asymmetry scores. Figure 3 illustrates group means for the cueing asymmetry scores.

**Table 3:** **Means and SDs (in brackets) for each condition and task; means and SDs for the CEs and cueing asymmetry scores have been derived from subtractions for each individual participant**.

	**RT task^a^**			**TOJ task^b^**		
	**Neglect**	**RHC**	**OHC^c^**	**Neglect**	**RHC**	**OHC^c^**
**LEFT TARGETS**						
Cued	790 (197)	1182 (1077)	447 (30)	0.35 (0.32)	0.17 (0.06)	0.08 (0.04)
Neutral	768 (204)	1189 (1046)	512 (42)	0.46 (0.30)	0.30 (0.07)	0.11 (0.08)
Uncued	944 (385)	1273 (1076)	487 (37)	0.54 (0.28)	0.31 (0.08)	0.28 (0.13)
CE	154 (259)	92 (106)	39 (17)	0.19 (0.20)	0.14 (0.09)	0.19 (0.13)
**RIGHT TARGETS**						
Cued	644 (166)	963 (602)	467 (40)	0.17 (0.20)	0.25 (0.15)	0.08 (0.04)
Neutral	714 (225)	1092 (895)	475 (30)	0.19 (0.19)	0.33 (0.13)	0.28 (0.03)
Uncued	685 (152)	1108 (818)	476 (47)	0.36 (0.24)	0.43 (0.15)	0.37 (0.04)
CE	42 (75)	145 (227)	9 (8)	0.19 (0.25)	0.18 (0.10)	0.29 (0.04)
**CUEING ASYMMETRY SCORE**						
	112 (218)	−53 (264)	30 (11)	0.0 (0.15)	−0.03 (0.11)	−0.10 (0.11)

a Raw units are mean RTs.

b Raw units are proportion of errors.

c Older healthy controls.

Because of the small number of participants in each group, we used non-parametric statistics to evaluate group differences in cueing asymmetry scores. While it appeared that the NEG group overall showed a rightward bias that was much greater than the OHC group, and that the RHC group showed a leftward bias, a Kruskal-Wallis one-way ANOVA revealed no significant difference in bias scores between the groups for the RT task (*H* = 0.08, *df* = 2, *p* > 0.9). To confirm that there was no difference between the stroke groups alone, we performed a Mann-Whitney U test on the mean bias scores for the NEG and RHC groups, also demonstrating no significant difference (*W* = 25, *n* = 12, *p* > 0.6).

**Figure 4:**
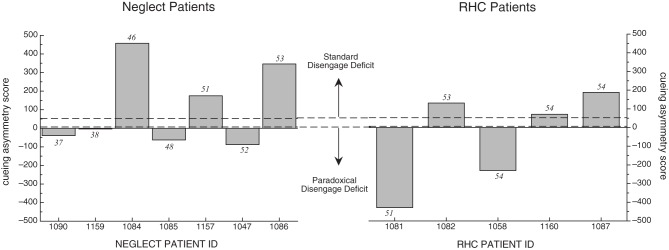
**Cueing asymmetry scores for NEG (left graph) and RHC group (right graph) in the RT task.** Patients are represented in order of the BIT Star Cancellation scores, from lowest to highest (individual BIT Star Cancellation scores are listed above or below the individual bars). Cueing asymmetry scores above zero represent a rightward bias. Note that the *x*-axis spaces patients equidistantly, and does not represent the listed BIT Star Cancellation scores on an ordinal scale. See text for details.

Because the mean cueing asymmetry scores for the NEG group appeared different from the RHC and OHC in the hypothesized direction, and because of the large variability, we examined cueing asymmetry scores for each individual participant in both the NEG and RHC groups. Figure 4 illustrates these individual cueing asymmetry scores, presented in order of their BIT Star Cancellation scores (indicated in the figures in italics). We also used the mean cueing asymmetry score +/−2 SD from the OHC group (indicated with dotted lines; 52 and 29, respectively) to determine which individual patients showed a right cueing asymmetry, indicative of a standard disengage deficit, or a left cueing asymmetry, suggesting a paradoxical disengage deficit. The graph reveals three interesting patterns:

1. All individuals in both stroke groups possessed cueing asymmetry scores that were +/−2 SD beyond the mean cueing asymmetry score of the OHC group, indicating that cuing effects for each patient were abnormal either on the left side *or* on the right side. That is, all patients in the RT task qualified for a disengage deficit label; however, they were almost equally likely to have a standard disengage deficit as they were to have a paradoxical disengage deficit.
2. Patients in the NEG group were no more likely than patients in the RHC group to have cueing asymmetry scores that put them in the range of a standard disengage deficit. In fact, more participants with neglect showed a paradoxical disengage deficit than a standard disengage deficit, albeit the paradoxical disengage deficits were smaller in terms of the absolute scores.
3. We found no relationship between neglect scores as derived from the BIT Star Cancellation task and the overall cueing asymmetry score in the RT task (*r* = 0.02, *df* = 10, *p* > 0.9). This was also true for the total BIT scores (*r* = 0.02, *df* = 10, *p* > 0.9) and the center of cancellation scores on the line cancellation subtest (see Rorden and Karnath, 2010; *r* = −0.02, *df* = 10, *p* > 0.9). That is, patients with clinical left neglect (indicated by low BIT Star Cancellation scores) did not necessarily have cueing asymmetry scores indicative of a standard disengage deficit. This was true for individuals in both the NEG and RHC groups (see Table 4 for the correlations and *p*-values comparing cueing asymmetry scores and scores from the BIT subtests and BIT subtest center of cancellation scores, using all of the stroke patients).

**Table 4:** **Pearson correlations for the subtests of the BIT with the cueing asymmetry scores for both the RT task and TOJ task, including patients from both the NEG and RHC groups**.

**BIT subtest**	**RT cueing asymmetry score**		**TOJ cueing asymmetry score**	
	**Correlation**	***p*-value**	**Correlation**	***p*-value**
Line cancellation	0.06	>0.8	0.07	>0.8
Line: CoC^a^	−0.06	>0.8	−0.07	>0.7
Letter cancellation	−0.01	>0.9	−0.11	>0.7
Letter: CoC^a^	−0.22	>0.4	0.04	>0.8
Star cancellation	0.01	>0.9	0.04	>0.8
Star: CoC^a^	−0.05	>0.8	0.24	>0.3
Line bisection	−0.23	>0.4	0.14	>0.6
Leftmost line bisection^b^	−0.27	>0.3	−0.15	>0.5
Figure copying	−0.17	>0.5	0.10	>0.7
Figure leftmost copy^b^	−0.43	>0.1	0.03	>0.9
Drawing	−0.01	>0.9	0.02	>0.9
Drawing leftmost item^b^	−0.42	>0.1	0.33	>0.2

a CoC, center of cancellation scores; see Rorden and Karnath, 2010 for score calculation.

b BIT subtests that were not predisposed to calculating a center of cancellation score were rescored to evaluate the left-most components.

### TOJ task results

To ensure that the cues in the TOJ task were effective at producing cueing, we examined PSS. A positive PSS indicates that the right-target would have to be presented before the left-target in order for a participant to experience them as being presented simultaneously; with the numerical value indicating how much of a lead the right-target would need (in ms). Three PSS scores were calculated per participant; one for trials on which a left cue was presented (left PSS), trials on which a neutral cue was presented (neutral PSS) and trials on which a right cue was presented (right PSS). Due to severe left neglect, we were unable to calculate reliable PSS scores for one neglect patient (ID 1159), so his data were excluded from the PSS cueing analysis. For the remaining participants, the mean PSS for these conditions was 295 ms (SD = 566), −58 ms (SD = 364), and −203 ms (SD = 413), respectively (see right graph, Figure 2). A one-way ANOVA revealed a significant effect of cue location [*F*(2, 26) = 5.96, *p* < 0.01, MSE = 154,041]. Table 3 includes the descriptive statistics for left- and right-side target CEs based on error rates as well as the cueing asymmetry scores (see section “*Methods of Analysis*”, above).

Just as in the RT task, we used non-parametric tests to evaluate group differences in cueing asymmetry scores for the TOJ task. A Kruskal-Wallis one-way ANOVA revealed no significant difference in cueing asymmetry scores between the groups (*H* = 1.72, *df* = 2, *p* > 0.4). To confirm that there was no significant difference among just the stroke groups, we performed a Mann-Whitney U test on mean cueing asymmetry scores for NEG and RHC groups, also demonstrating no significant difference (*W* = 21, *n* = 12, *p* < 0.64). Figure 3 (right graph) illustrates group means for the cueing asymmetry scores for the TOJ task.

**Figure 5:**
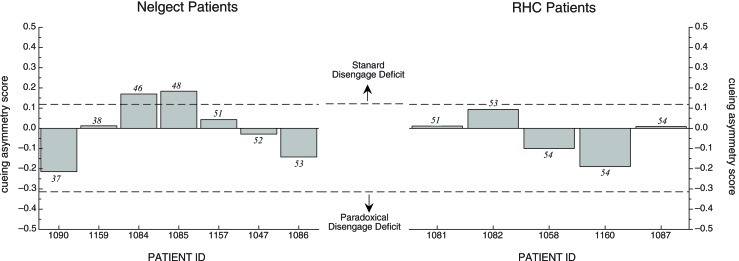
**Cueing asymmetry scores in the TOJ task for Neglect patients (left graph) and RHC patients (right graph) plotted in order of each patient's BIT Star Cancellation score (see text for details).** Dotted lines represent +/− 2SD of mean OHC cueing asymmetry score.

Because TOJ cueing asymmetry scores had a finite range (1 to −1), and because the mean cueing asymmetry scores for each group were relatively small in comparison to this range, we were not compelled to examine the individual cueing asymmetry scores. However, to be consistent with the analysis in the RT task, we plotted the individual scores for each patient group in Figure 5 in order of their BIT Star Cancellation scores (indicated in italics). We again used the mean cueing asymmetry +/−2 SD from the OHC group (indicated with dotted lines; 0.12 and −0.31, respectively) to define a standard disengage deficit or a paradoxical disengage deficit. The graph reveals two interesting patterns:

1. Most individuals in both groups were within a normal range, as defined by the mean cueing asymmetry score of the OHC group +/−2 SD.
2. The two individuals with neglect whose cueing asymmetry scores were outside the normal range (OHC) and in the range of a standard disengage deficit did not have the most severe clinical neglect as determined by their BIT Star Cancellation scores. Indeed, there was no overall relationship between cueing asymmetry scores for the TOJ task and any of the BIT subtests (correlations and *p*-values are listed in Table 4).

### Post-hoc analyses

To ensure the results were not due to a lack of counterbalancing the keys, we looked at responses as a function of response keys (“8” vs. “2”). For RT in the RT task, there was no main effect of key across all participants using a within-subject one-way ANOVA. There was also no interaction between key and participant group in a mixed ANOVA. This was also true for the TOJ task.

**Figure 6:**
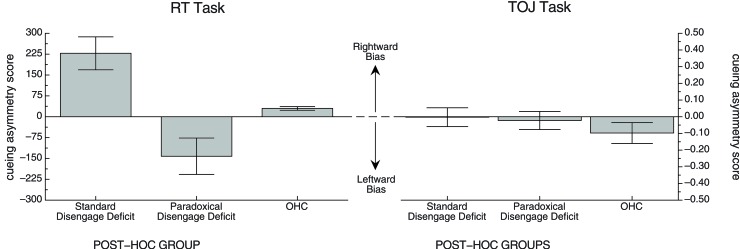
**Mean cueing asymmetry scores in each task for the post-hoc groups.** OHC group is included for reference. See text for details.

The correlation between the cueing asymmetry scores in two tasks, including all participants in all groups, was small and not significant (*r* = 0.08, *df* = 13, *p* > 0.7). We wanted to confirm that there was no relationship between cueing asymmetry scores in the RT task and TOJ task. To this end, we created two post-hoc groups of participants from the stroke population in our study: a Standard Disengage Deficit Group and a Paradoxical Disengage Deficit Group. These groups were chosen *based on each patient*'*s cueing asymmetry score from the RT task only*. As one would expect, a Mann-Whitney U test revealed a significant effect of the post-hoc grouping on cueing asymmetry scores (*W* = 36, *n* = 12, *p* < 0.01). We then kept the groups the same to examine whether individuals who showed a standard disengage deficit in the RT task also show a standard disengage deficit (or even a bias in that direction) in the TOJ task. A Mann-Whitney U test revealed no significant difference between the post-hoc groups on cueing asymmetry scores in the TOJ task (*W* = 20, *n* = 12, *p* > 0.8). Figure 6 illustrates the mean cueing asymmetry scores for these post-hoc groups for both tasks. The TOJ graph nicely illustrates that there appears to be no difference between groups. Indeed, neither the Standard Disengage Deficit Group nor the Paradoxical Disengage Deficit Group showed a cueing asymmetry score different from zero (*x* = −0.003, *df* = 5, *p* > 0.9, and *x* = −0.02, *df* = 5, *p* > 0.6, respectively). The OHC group is included in the figure for reference.

## Discussion

In the current study we sought to examine whether the disengage deficit typically observed in an RT task in patients with unilateral neglect might also be observed in the same patients using a TOJ task with spatial cueing. To this end, we compared performance on a standard speeded RT cueing task to a TOJ-with-cueing task that were equated for stimuli and response choice discrimination. It should be noted that for both tasks the peripheral cues were uninformative. As noted by Losier and Klein (2001) when neglect patients are subjected to a typical spatial cuing paradigm, the disengage deficit in RT is robust when peripheral cues are used (whether or not these cues are informative) while the disengage deficit is small to absent following purely endogenous cuing (informative central arrow cues).

Performance on both tasks showed significant cueing effects as expected and validated the cueing manipulation. Across both tasks, however, there were three major surprising and noteworthy patterns of results:

1. Few stroke patients showed a disengage deficit in the TOJ task and cueing asymmetry scores in the TOJ task were not consistent with the cueing asymmetry scores in the RT task, either in a correlational analysis, or by post-hoc grouping.
2. In the RT task typically used to demonstrate a standard disengage deficit, stroke patients were almost equally likely to show a standard disengage deficit (indicative of a spatial bias in favor of the ipsilesional side of space) as they were to show a paradoxical disengage deficit (indicative of a spatial bias in favor of the contralesional size of space).
3. There was no relationship between the size and/or direction (i.e., whether it was standard or paradoxical) of the speeded disengage deficit and neglect severity. Indeed, patients who did not meet the criteria for clinical neglect (i.e., RHC) were just as likely to have a disengage deficit as patients who did meet the criteria for clinical neglect (i.e., NEG).

The cueing asymmetry scores in the two tasks were not consistent with each other, and indeed patients seemed to have very low cueing asymmetry scores in the TOJ task. This inconsistency would indicate that the disengage deficits evident in the RT task are a manifestation of the task demands rather than a general attentional state in patients with or without neglect. One possibility is that the differences in task demands elicited different mental sets; in the RT task the action system needed to be rapidly recruited, while in the TOJ task it did not.

Goodale and Milner (1992); Milner and Goodale (2008) proposed an influential model of vision that divides visual processing into two functional streams. The dorsal stream is involved in the use of vision for action; this stream controls detailed programming of online movements using bottom-up inputs from the retina to determine the specific parameters for movement. The ventral stream is involved with vision for identification; this stream enables the perception and identification of objects and their spatial relations. Knowing in advance that they would be required to execute a speeded motoric response in the RT task involving a key choice could have set the participants up to engage their dorsal stream. The cue may have provided an exogenous trigger for the initiation of a visually-guided motor plan, which would have to be inhibited in favor of a new plan triggered by the target. However, neglect patients often show motor perseveration—that is, a continuation of a behavior after a change in task demands (Kim et al., 1999). For example, neglect patients frequently mark individual stars repeatedly in the BIT star cancellation task, even while ignoring all of the stars in their neglected field. Engagement of the dorsal stream coupled with motor perseveration would preferentially impact reaction times in the RT task, but not accuracy in the TOJ task.

Another surprising finding, however, was that a direction of the patient's deficit in the RT task was not necessarily predicted by the location of their lesion or the side of space they were prone to neglect in standard tests of neglect. About half of the patients with right hemisphere damage, irrespective of whether they met the criteria for contralesional neglect or not, showed a disengage deficit in the RT task that was in *favor* of their contralesional side of space (i.e., demonstrating a paradoxical disengage deficit). The presence of a paradoxical disengage deficit as seen in our study has also been reported by others (e.g., Sacher et al., 2004 in their individual analyses). One reason for this pattern may be due to compensatory effects (Robertson et al., 1994; Dove et al., 2007). That is, patients who have contralesional neglect and are aware of their deficit may overcompensate for their spatial bias by making an effort to direct their attention leftward. Compensatory strategies for a task involving a time-pressured response coupled with motor perseveration might explain the paradoxical disengage deficits that were observed on some of the patients tested, although this compensation was not evident in the TOJ task. The role of compensatory strategies in neglect recovery and interaction with task demands has been highlighted in recent papers and reviews (e.g., Manly et al., 2002; Bonato et al., 2010; Bonato, 2012).

The fact that the standard disengage deficit was not consistent in the NEG group, and was also present in some of the RHC group is perhaps not surprising. Previous studies have shown that patients who have right hemisphere damage but who do not meet the criteria for clinical neglect might also have a standard disengage deficit, suggesting that some experimental tasks, including the Posner spatial cueing RT task that we used, might be more sensitive at detecting behavioral neglect than the standard clinical tests of neglect (Rengachary et al., 2009). In their meta-analysis, Losier and Klein (2001) found that the standard disengage deficit was significantly more severe in patients with neglect and, among those with neglect there was a significant correlation between neglect severity and the size of the disengage deficit. Neither of these patterns held up in our group of patients; however, this pattern is itself not entirely consistent in the literature. For example, Sieroff et al. (2007) found that there was no relationship between the magnitude of the disengage deficit and neglect severity in a group of patients defined in a similar way as our study. The lack of a group correlation between the disengage deficit and neglect severity, or the dissociation between the presence of a disengage deficit and neglect symptoms on clinical tests in individual patients has also been reported in several studies (e.g., Sacher et al., 2004; Olk et al., 2010; Bonato et al., 2012). Thus, further examination of what other variables may underlie the disengage deficit pattern appears warranted.

One explanation for our deviation from this pattern could be the variability of the data itself. The mean cueing asymmetry score for the NEG group was not significantly different from zero, and the only significant effects were found in the post-hoc groups, which is no surprise. What is noteworthy is that even when we grouped the patients according to whether they showed a standard disengage deficit in the RT task, they did not show a corresponding disengage deficit on TOJ task.

The performance of our patient participants on the RT task is more variable between-subject than the representative literature (cf., Losier and Klein, 2001), but there are several reasons that may explain this difference. First, we used relatively unrestricted inclusion criteria for our neglect patients and our individual analyses may have identified more variability than is normally included in small group studies. Second, researchers often report data in aggregate form, which may obscure individual differences among patients, and hide patients who lack the standard disengage deficit. We specifically examined the individual patients to determine how variable the disengage deficit was. We were surprised by the variability; however, we also felt that the extent and direction of the variability itself was interesting and important to report. Third, patient or methodological differences in the current study may have led to these findings. We chose to use a discrimination task in the cuing paradigm, in order to directly match the task requirements from the TOJ task. Since most cuing tasks use a detection response, this difference may have created some of the variability. Further investigation into the impact of type of task processing on the spatial cuing paradigm may help to resolve this issue. In any event, the disengage deficit has been regarded as a better test of neglect than the standard clinical tests, but if patients only show the effect under a very narrow range of conditions, the disengage deficit may not be an effective explanation or marker of neglect. Finally, and possibly most critically, the variability of performance among neglect patients in a spatial cueing paradigm may be under-reported due to a publication bias. Authors may shelve studies in which the patients are highly variable because the data don't conform to the author's theoretical framework, or may be rejected by reviewers because the data don't conform to the literature. However, every patient is providing valuable data, irrespective of one's theoretical framework. It is important that a literature represents the population it is studying, and in this case that population is highly variable.

Whatever the reason, the results of our study show that the disengage deficit is neither necessary nor sufficient to produce neglect. Not necessary because patients with left neglect might show no disengage deficit or a paradoxical disengage deficit; not sufficient because some patients without classic neglect as seen on paper and pencil tasks (i.e., RHC patients) show the standard disengage deficit. Our finding that the disengage deficit is not consistent across well-matched tasks (RT cuing vs. TOJ) also suggests that the disengage deficit is perhaps not the unifying explanation of neglect that some researchers hoped it would be (Adair and Barrett, 2008). In addition, our findings suggest that a better understanding of contributory factors that can influence visuo-spatial responding (e.g., non-spatial attention deficits, compensation strategies, and the role of task demands and manual responses) appears necessary to further advance theories of the basic mechanisms underlying spatial neglect.

### Conflict of interest statement

The authors declare that the research was conducted in the absence of any commercial or financial relationships that could be construed as a potential conflict of interest.

## Footnotes

^1^Losier and Klein's (2001) meta-analysis included studies that used both predictive and non-predictive cues. In the current study, we used non-predictive cues in both tasks; however, Losier and Klein found that the disengage deficit is robust with peripheral cues whether or not they are informative.

## Appendix

**Table A1:** **Behavioral inattention task subtest scores**.

**Subject code**	**Group**	**Line cancellation (center of cancellation)**	**Letter cancellation (center of cancellation)**	**Star cancellation (center of cancellation)**	**Copying (only left errors)**	**Line bisection center of cancellation (left-most line only)**	**Drawing (only left errors)**
1047	NEG	36 (0.00)	40 (0.00)	49 (0.01)	4 (4)	0.07 (0.11)	3 (3)
1084	NEG	36 (0.00)	37 (0.03)	46 (0.05)	3 (3)	−0.18 (−0.16)	2 (−)
1085	NEG	35 (0.00)	39 (0.00)	48 (0.02)	2 (4)	0.05 (0.26)	1 (2)
1086	NEG	36 (0.00)	33 (−0.06)	52 (0.01)	3 (3)	0.11 (0.15)	3 (3)
1090	NEG	36 (0.00)	34 (0.15)	37 (0.32)	2 (2)	−0.06 (−0.04)	1 (1)
1157	NEG	36 (0.00)	40 (0.00)	51 (0.04)	1 (1)	−0.10 (0.01)	1 (1)
1159	NEG	30 (0.19)	22 (0.27)	39 (0.17)	2 (3)	0.25 (0.48)	3 (3)
1058	RHC	36 (0.00)	38 (0.00)	54 (0.00)	2 (4)	−0.10 (−0.08)	3 (3)
1081	RHC	36 (0.00)	35 (0.06)	51 (0.01)	4 (4)	0.05 (0.18)	3 (3)
1082	RHC	36 (0.00)	40 (0.00)	53 (0.00)	3 (3)	−0.06 (0.02)	3 (3)
1087	RHC	36 (0.00)	34 (0.02)	54 (0.00)	2 (3)	0.04 (0.10)	3 (3)
1160	RHC	36 (0.00)	38 (−0.05)	54 (0.00)	4 (4)	−0.02 (−0.07)	3 (3)
1162	OHC	36 (0.00)	40 (0.00)	54 (0.00)	4 (4)	0.00 (0.01)	3 (3)
1163	OHC	36 (0.00)	40 (0.00)	54 (0.00)	4 (4)	0.02 (0.05)	3 (3)
1164	OHC	36 (0.00)	40 (0.00)	54 (0.00)	4 (4)	−0.01 (0.01)	3 (3)

NEG, neglect patient; RHC, right hemisphere control patient; OHC, older healthy control participant.
